# Thermal blanketing by ivy (*Hedera helix* L.) can protect building stone from damaging frosts

**DOI:** 10.1038/s41598-018-28276-2

**Published:** 2018-06-29

**Authors:** Martin A. Coombes, Heather A. Viles, Hong Zhang

**Affiliations:** Oxford Rock Breakdown Laboratory (OxRBL), School of Geography and the Environment, University of Oxford, South Parks Road, OX1 3QY Oxford, UK

## Abstract

The impact of plants growing on buildings remains controversial, especially for vulnerable historic walls and ruins requiring on-going conservation. English ivy (*Hedera helix* L.) can cause considerable damage where it is able to grow into deteriorating masonry, yet in some circumstances it may be protective. Here we focus on the potential of ivy to buffer damaging thermal cycles and frost events that can contribute to the deterioration of masonry materials. On limestone masonry test walls in central Southern England (Wytham near Oxford, UK), ivy foliage had a significant influence on stone-surface freezing regimes. Over two successive winters (2012/13 and 2013/14) the frequency of freezing events under ivy was reduced on average by 26%, their duration by 34% and their severity by 32%. A subsequent laboratory simulation showed that stone mass loss, surface softening, and textural development were all significantly reduced under an ‘ivy covered’ thermal regime. Cautious extrapolation indicates that ivy can reduce frost-driven granular-scale decay of limestone by the order of 30 g m^−2^ yr^−1^, depending on the local freezing regime. Whilst the capacity of ivy to cause damage should not be underplayed, vertical greenery can aid heritage conservation efforts by mitigating specific environmental threats.

## Introduction

The potential for ivy and other higher plants growing on masonry structures to cause damage in some circumstances is unquestionable^1–6^. For historic structures, greatest damage is associated with stems growing into existing defects (i.e., holes, cracks, deteriorating mortar etc.), where very thick (older) stems grow under wall foundations, and where the plant produces ‘true’ roots within the fabric of walls^7,8^. Whilst these damaging effects do not always occur, ivy has gained widespread reputation as a considerable nuisance for historic buildings and for those tasked with their management and conservation. Very often active measures are taken to stop ivy from growing, and where it has become established, the decision is often made to remove it entirely.

Removal is, however, often difficult, time consuming and expensive, and in some cases attempts to remove well-established growth will have disastrous consequences for the underlying structure^8^. There are also strong arguments against removal given its considerable contribution to biodiversity^9–11^ and – some would argue – aesthetic appeal^12–14^. This is especially true in the case of ruined sites^15^ and at a time when ‘greening’ of the built environment to maximise ecological and social value is high on the policy agenda^16–18^.

Accepting its potential to cause damage, there is evidence to suggest that in some cases a cover of ivy can be beneficial for historic walls, and that costly removal is unnecessary or entirely inappropriate. For example, where underlying masonry is in a good state of repair and appropriate efforts are made to trim or restrict growth away from gutters and roofs^19^, the risk of ivy stems penetrating into a structure is negligible^8^; ivy attaches by means of surficial aerial rootlets and stems have no capacity to ‘bore’ into construction materials unless defects already exist^8,20–22^. Where masonry is sound, any perceived risk of structural damage could be outweighed by other beneficial effects. This may include shielding walls from driving rain^7,23,24^ and filtering particulate pollution linked to chemical and aesthetic degradation of vulnerable stone^25^. In addition, a cover of English ivy has been found to reduce the range and variability of wall-surface microclimates over diurnal, seasonal and annual timescales, at a number of historic sites in England^26^. Influences on surface wetting may also have consequences for thermally-driven deterioration at the event-scale^27^. Because of its impacts on temperature regimes, weathering theory suggests that ivy cover should reduce deterioration associated with repeated warming-cooling and expansion-contraction of stone and mortar^27–30^.

In winter, the ‘thermal blanket’ effect of ivy (and other vertical greenery) may be particularly important for vulnerable stone, by buffering freezing events that otherwise occur on exposed walls^24,26,31^. For historic masonry, especially porous stone, brick and mortar, damage from freeze-thaw is recognised as a significant environmental threat^32–34^. Whilst climate change is likely to reduce the occurrence of freezing in some parts of Europe^35^, it might enhance it in other areas, and recent modelling indicates that the probability of severe and persistent cold winters may increase over the next few decades^36–38^. Furthermore, assuming generally wetter winters in the UK under future climate scenarios, the likelihood of masonry being wet when freezing does occur (even if this is less frequent) is greatly increased^39–42^.

Freezing, ice segregation and the formation of damaging ice crystals in rock, stone and other masonry materials is a threshold-driven process^34,43,44^. Factors having even a slight influence on wall microclimates (such as vegetation cover) can therefore have significant consequences for frost-driven deterioration. Complicating this is the fact that—in addition to frequency of occurrence—the severity, rate and duration of freezing have bearing on the potential for damage^42,45–47^. Whilst *H. helix* is intolerant of persistent cold (sustained periods of −2 °C or below) it can tolerate significant cold snaps down to −25 °C^48^. Furthermore, as an evergreen, ivy retains its foliage during the coldest months of the year. Existing studies provide a strong evidence base for a protective mechanism with respect to frosts, yet these effects are likely complex and a more detailed assessment of ivy’s influence on the nature of frost events is missing. For example, it is not known how the efficacy of frost buffering varies for events of differing severity, or whether the effect is consistent between wall aspects. Most importantly, it remains untested whether thermal buffering (including frosts) leads to reductions in rates of physical deterioration of masonry materials.

To address these gaps, we undertook a two-phase study. First, continuous monitoring (2012 to 2014) was undertaken on limestone (Elm Park) masonry test walls at Wytham Woods, 4 km north-west of Oxford, UK (51°46′25 N, 1°19′23 W). Data were used to assess the influence of ivy on the frequency, severity and duration of frost events. A comparison between four aspects (north-, east-, south- and west-facing walls) was also undertaken. Second, results from the field monitoring phase were used in combination with meteorological data to test the influence of frost buffering by *H. helix* on the physical deterioration of stone. This was done by exposing samples to ‘ivy covered’ and ‘exposed’ thermal regimes in accelerated laboratory weathering simulations, and recording relative mass loss, surface softening and textural development.

## Results

### Test wall monitoring

**Figure 1:**
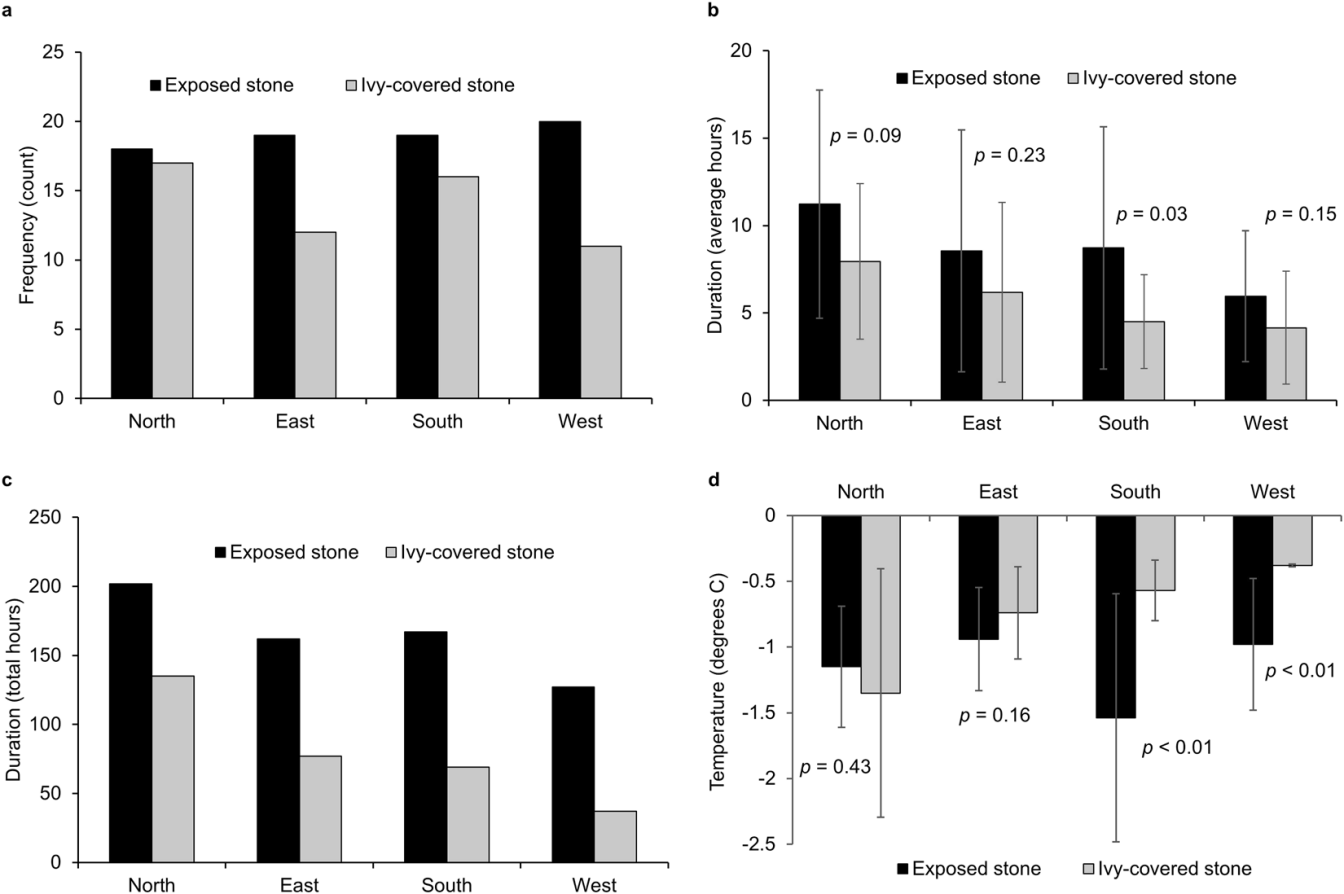
Frost (**a**) frequency, (**b**) duration, mean ± SD, (**c**) total frozen hours, and (**d**) severity, mean ± SD, on exposed and ivy covered sections of north-, east-, south- and west- facing test walls (winter 2012/13 and 2013/14), Wytham Woods, Oxfordshire. Significance of t-test comparisons between exposed and ivy covered sections of wall are shown for duration (**b**) and severity (**d**) of all recorded events.

Over the two winters monitored during the study, 76 individual freezing events were recorded on exposed sections of wall compared to 56 events on ivy covered sections (χ^2^[1, N = 132] = 3.03, *p* = 0.08). Overall, frost frequency was reduced by 26% but the magnitude of this effect varied between aspects (Table 1, Fig. 1a). On west- and east-facing walls the frequency of freezing was reduced by 45% and 37%, respectively, compared to 16% on the southern aspect and 6% on the northern aspect (χ^2^[3, N = 20] = 8.00, *p* = 0.05).

**Table 1:** Characteristics of freezing events recorded on test walls during winter (cumulative for 2012/13 and 2013/4) for exposed and ivy covered sections.

	North		East		South		West	
	Exposed	Ivy	Exposed	Ivy	Exposed	Ivy	Exposed	Ivy
Frequency *Influence of ivy cover*	**18**	**17**	**19**	**12**	**19**	**16**	**20**	**11**
	−*6%*		−*37%*		−*16%*		−*45%*	
Duration (hours) *Influence of ivy cover*	**11.2**	**7.9**	**8.6**	**6.2**	**8.7**	**4.5**	**6.0**	**4.2**
	−*30%*		−*28%*		−*48%*		−*30%*	
Severity (degrees Celsius) *Influence of ivy cover*	−**1.2**	−**1.4**	−**0.9**	−**0.7**	−**1.5**	−**0.6**	−**1.0**	−**0.4**
	+*17%*		−*21%*		−*63%*		−*61%*	

‘Frequency’ is the number of fluctuations below and above 0 °C; ‘duration’ is average time (in hours) temperatures fell below 0 °C per freezing event; ‘severity’ is the average minimum temperature (in °C) reached per freezing event. The influence of ivy relative to exposed stone (% difference) is indicated.

**Figure 2:**
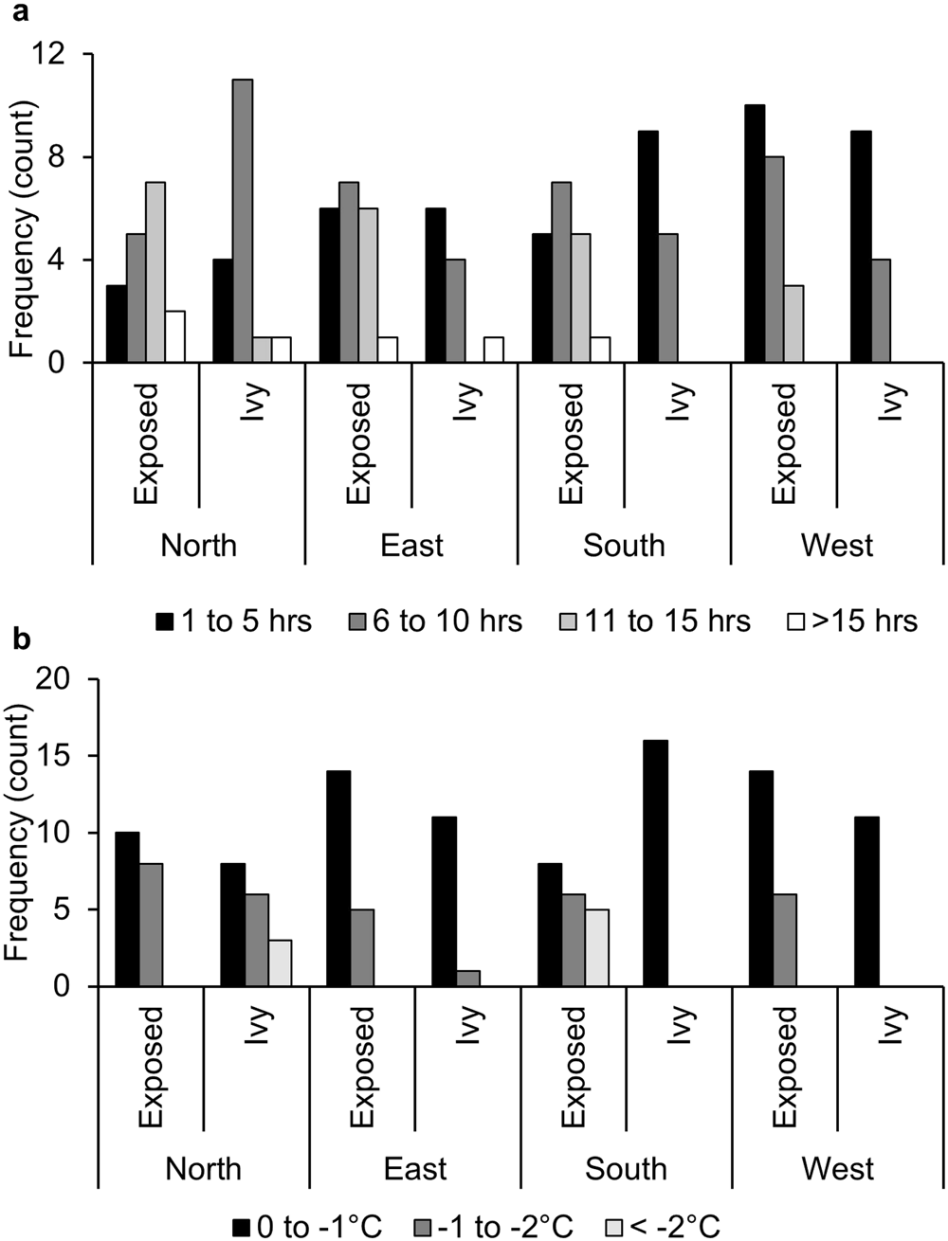
Number of frost events of differing (**a**) duration, hours, and (**b**) severity, °C, recorded on test walls with and without a cover of ivy (winter 2012/13 and 2013/14), Wytham Woods, Oxfordshire.

The durations of freezing events were also reduced under ivy on all aspects (Fig. 1b). The overall influence was a reduction from an average of 8.5 hours on exposed stone to 5.8 hours on ivy covered stone (t[129, N = 132] = 2.95, *p* < 0.01). Figure 2a summarises the durations of frost events recorded on each wall. On east- and west-facing aspects, the number of events of each duration class were always the same or lower under ivy compared to exposed stone. On north- and south-facing walls, the number of events lasting 11 hours or more were also reduced under ivy, but shorter-duration events were more frequent (Fig. 2a). This implies a general shift from longer-duration to shorter-duration freezing events under ivy. The impact on freezing duration was further demonstrated by the cumulative total number of frozen hours, which was significantly reduced under ivy (by an average of 54%) irrespective of aspect, (χ^2^[6, N = 976] = 17.29, *p* < 0.01), Fig. 1c.

The average severity of freezing events recorded during the study period was −1.15 ± 0.65 °C on exposed stone compared to −0.8 ± 0.68 °C on ivy covered stone. Although small in real terms, this difference was statistically significant (t[129, N = 132] = 2.84, *p* = 0.05) but did vary between aspects; frost severity was reduced under ivy on the east-facing (*p* = 0.16), south-facing (*p* < 0.01) and west-facing (*p* < 0.01) walls, but was higher (not significantly) on the north-facing wall (*p* = 0.43), Fig. 1d. The overall pattern of frost severity also varied between exposed and ivy covered sections of wall (Fig. 2b); occurrence of low-severity events (0 to −1 °C) was the same (46 in both cases) but ivy covered stone experienced fewer events reaching −2 °C and below. This reflects an overall shift from higher to lower severity events under a cover of ivy.

### Weathering simulations

**Figure 3:**
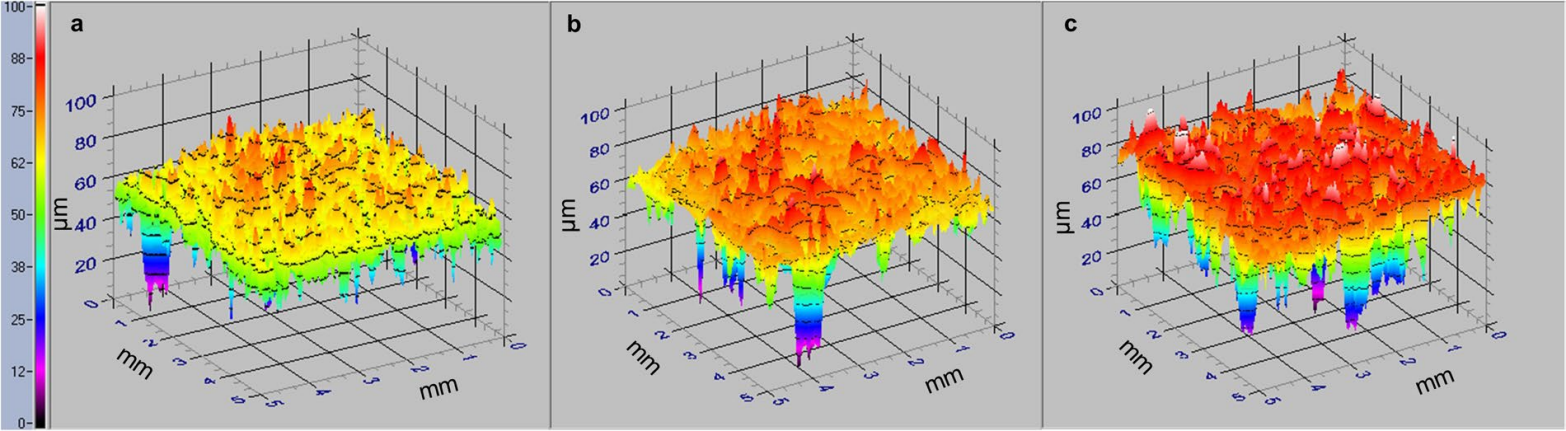
3D models derived using the TRACEiT® optical scanner for surfaces of Elm Park Limestone exposed to: (**a**) no weathering (control); (**b**) an ivy covered frost weathering regime and (**c**) an exposed stone (no ivy) frost weathering regime. In each case a 5 × 5 mm area is shown, with vertical height (Z) deviation represented in colour shading between 0 and 100 µm.

All limestone samples lost weight, softened and became rougher during the frost weathering simulations (Table 2). However, there were marked differences in the response of samples subjected to the different thermal regimes. Direct breakdown (mass loss) was on average 27% lower for stone exposed to an ivy covered regime compared to the exposed regime (t[8, N = 10] = 3.63, *p* = 0.01), Table 2. Softening of the stone surface (measured as a reduction in Equotip Leeb hardness) was similarly reduced for the ivy covered regime, by an average of 64% relative to exposed stone conditions (t[8, N = 10] = 2.89, *p* = 0.02). Samples subjected to the exposed stone regime were also significantly rougher by the end of the simulation (t[48, N = 50] = 13.42, *p* < 0.01) indicating a greater degree of surficial breakdown (Table 2 and Fig. 3). There were no changes in weight, hardness or surface roughness of control samples.

**Table 2:** Indicators of breakdown (mass loss, surface softening and roughness) for Elm Park Limestone after being subjected to ‘exposed stone’ and ‘ivy covered’ frost weathering simulations (mean ± SD, n = 5).

	Mass loss (g)	Surface softening (*L*)	Surface roughening (Ra, µm)
Exposed stone regime	−0.262 ± 0.04	−29.52 ± 8.32	+4.43 ± 0.36
Ivy covered regime	−0.190 ± 0.03	−10.77 ± 11.90	+1.43 ± 0.72
Influence of ivy thermal buffering	27% less breakdown (*p* < 0.01)	64% less softening (*p* = 0.02)	68% less roughening (*p* < 0.01)

**Figure 4:**
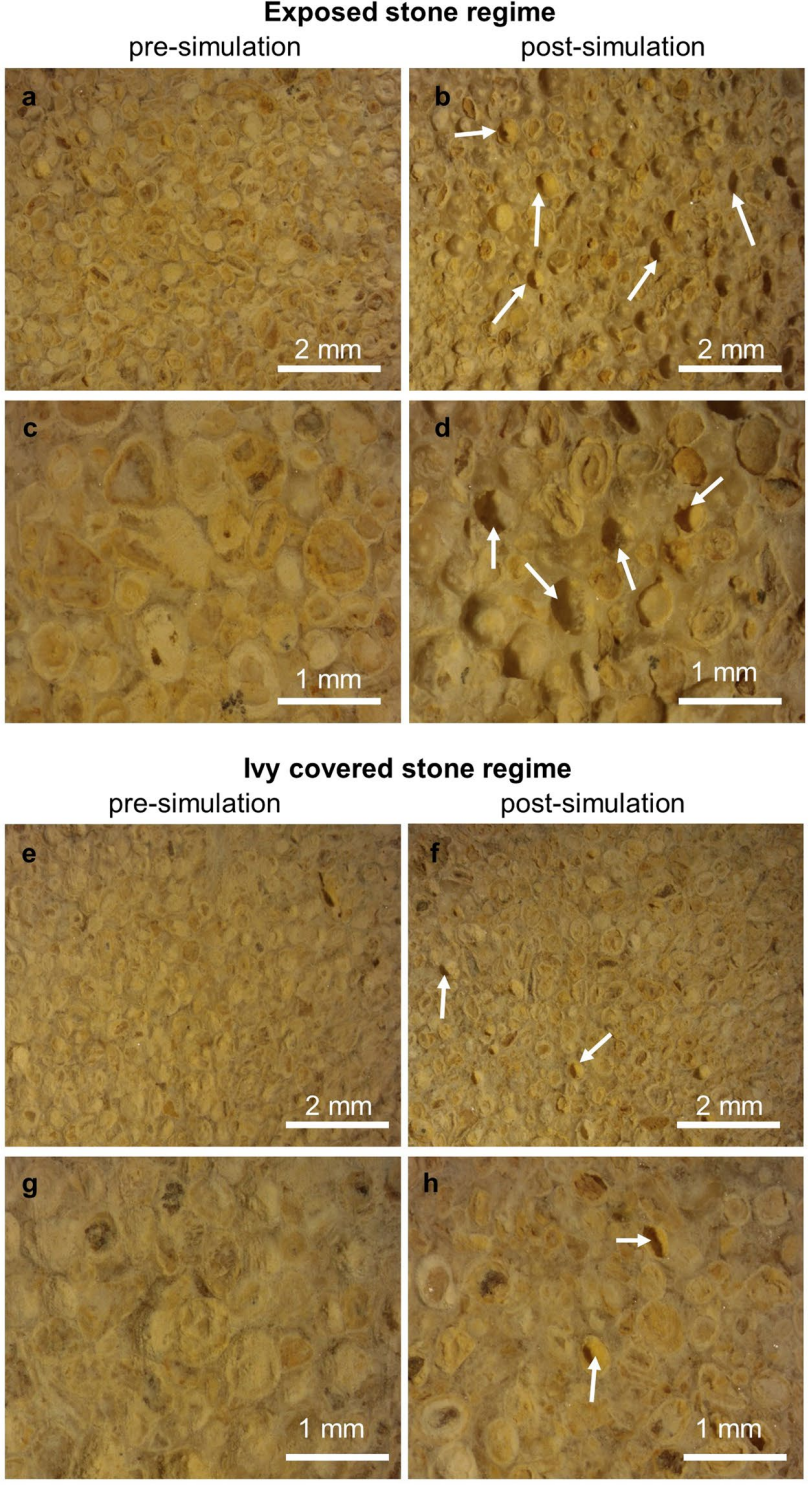
Indicative microscope images of Elm Park Limestone before and after frost weathering simulations for ‘exposed stone’ (**a**-**d**) and ‘ivy covered’ regimes (**e**-**f**). Two magnifications, scale as shown. Areas of ooid loss are indicated.

Optical microscopy revealed that breakdown was via surficial granular disintegration; post-simulation samples showed clear evidence of ooid liberation from the surfaces onto which moisture had been applied (Fig. 4). The extent of granular loss was visibly greater for samples subjected to the ‘exposed’ thermal regime (Fig. 4), corroborating the relative changes in physical properties summarised in Table 2.

## Discussion

We found that freezing events were less frequent, shorter in duration, and less severe under *H. helix* relative to exposed stonework. There was some variation in the nature of these effects between wall aspects, likely reflecting differences in insolation regimes (Table 1, Figs 1 and 2). Ivy buffered freezing events down to −3.5 °C in the field. It is not yet possible to state how the efficacy of buffering will vary for higher-magnitude (more severe) events. For example, the ability of ivy to sustain wall surface temperatures above freezing (and thus out of the ‘frost cracking window’^47^) may be reduced/ negated when air temperature falls below some threshold value. This has important implications when considering, for example, how the frost-buffering potential of vegetation varies geographically as well as the mode of breakdown that may be being prevented, e.g., frost shattering or ice segregation by low-frequency, high-magnitude events, or progressive subcritical fatigue by high-frequency, low-magnitude events. Associations between these two different modes of breakdown, whereby progressive subcritical crack propagation can gradually lead to failure^49^, implies that ivy’s influence on both is of potential significance. Sternberg *et al*.^26^ reported that the greatest difference in daily minimum temperatures (between exposed and ivy covered walls) occurred at their northern-most study site at Byland in Yorkshire. Bolton *et al*.^31^ also found that thermal insulation by *H. helix* on walls in northern England was most pronounced on the coldest days. This indicates that vegetation may have the greatest protective benefit in the coldest parts of the UK, such as northern England and Scotland, and during the coldest conditions when the potential for ‘shock’ modes of ice-driven damage (i.e., frost shattering, ice segregation and spalling) is greatest^50^.

Whilst an absence of very severe freezing events at the test walls precluded any direct assessment of protection against ‘shock’ modes of breakdown, ivy’s clear moderating influence on temperature is of significance for building conservation. As found in other studies^26,31^, potential damage to masonry caused by repeated but less-severe thermal fluctuations was reduced under ivy. Across four wall aspects, we found that summertime thermal maxima were 17% lower and winter thermal minima were 40% higher under ivy. Over the course of a whole year (April 2013 – April 2014), this represented a reduction in the average diurnal temperature range of more than 40% relative to exposed stone (4.3 °C compared to 7.9 °C, respectively). These effects will limit the extent of repeated, thermally-related expansion and contraction of masonry materials that can lead to physical fatigue^8,24^.

Establishing clear links between thermal ‘fatigue’ and ‘shock’ is challenging, but they certainly do not operate in isolation^27^. Progressive weakening via any small, but regular thermal stresses can lead to eventual failure via propagation of subcritical cracks^47,49^. Indeed, given that fracture toughness (the ability of a material to resist fracture) is a product of both stress magnitude and crack length, low-magnitude, but frequently occurring stress events can lengthen cracks and thereby lower the required stresses for subsequent failure^49^. By reducing the frequency, duration and severity of lower-magnitude freezing events, as well as the occurrence and amplitude of thermal cycling over a range of timescales, our observations indicate that ivy can reduce the vulnerability of masonry to fatigue-driven deterioration as well as catastrophic modes of breakdown when very severe events occur^51^. Fracture mechanics theory is consistent with the generally accepted view that minimising the variability of environmental conditions that historic structures are exposed to will be beneficial for long-term conservation. Data collected at the test walls show very clearly that, in the right circumstances, ivy can play a role in achieving this.

Our laboratory experiments show for the first time that thermal buffering by ivy can reduce stone deterioration, expressed as a reduction in the rate of physical loss of material, surface softening and textural development (Table 2). The numbers of freezing events used in our simulations (see Table 3) fall within the typical range of annual ground-frost days recorded in southern England by the UK Met Office. Cautious extrapolation therefore suggests (based on the relative changes in Table 2) that frost buffering by a cover of ivy can reduce deterioration of (freshly exposed) limestone by the order of 30 g m^−2^ yr^−1^. This is comparable with deterioration rates reported for calcareous stone exposed at a range of urban and rural sites across the UK^52^ and Europe^53^. This implies that, in frost-prone climates, the relative significance of thermal buffering by vegetation is comparable to surface losses resulting from other weathering processes such as dissolution. Importantly, our measurements only accounted for surficial granular-scale decay (Fig. 4), excluding any additional protection that ivy may provide from infrequent but very severe freezing events. It should also be acknowledged that the protective influences of vegetation may be more/less relevant for stone (and other building materials) with different physical properties, such as strength and pore-size distribution, that can influence susceptibility to thermally-driven breakdown.

**Table 3:** Freezing regimes used to simulate frost weathering in the laboratory.

	Exposed stone regime	Ivy covered regime (as adjusted based on test wall data)
Frequency of freezing cycles	4 cycles per 48 hours	3 cycles per 48 hours
Duration of freezing cycles	5.5 hours freezing *transition (0.5 hours)* 5.5 hours thawing *transition (0.5 hours)*	3.5 hours freezing *transition (0.5 hours)* 7.5 hours thawing *transition (0.5 hours)*
Severity of freezing cycles	Freezing at −5 °C Thaw at +9 °C	Freezing at −2 °C Thaw at +8 °C

By altering physical material properties, repeated thermal cycling (as measured at the test walls) and freezing and thawing (as simulated in our laboratory experiments) can render masonry materials more vulnerable to future thermal stresses and frost, as well as other mechanical and chemical weathering processes^51,54,55^. This is of particular significance for historic buildings and ruins that are often vulnerable to on-going and sometimes rapid decay, especially where funds available for interventive repair and maintenance are limited^8^. Further experiments employing more severe freezing conditions would be beneficial, as would experiments using larger sample blocks for which thermal gradients and ice segregation mechanisms may be more important^56^. Collection of field data from areas with severe freezing regimes would be extremely beneficial in this regard. There is also a need to extend observations to other masonry materials (including brick and mortar) as vulnerability to frost damage can vary considerably^57^, meaning the relative benefit of ivy cover may vary.

By varying temperature regimes during the two laboratory simulations (ivy covered and exposed stone) but applying moisture in exactly the same way, we found that ivy’s thermal buffering influence alone can reduce physical deterioration of limestone. As ivy foliage can also act as a rain shield, the measured effects are considered to be very conservative. The existence of critical saturation thresholds for freeze-thaw weathering is debated, but all ice-driven modes of mechanical breakdown will be greatly reduced where moisture supply is limited^27,58–60^. In this respect, the vulnerably of masonry to frost damage may be further reduced by vegetation acting as a barrier to moisture during precipitation events^23,61^.

## Conclusions

A cover of English ivy reduced the frequency, duration and severity of freezing temperatures and of diurnal thermal cycling at the surface of limestone masonry walls in central southern England. Whilst there was some small variation between aspects, the overall trend was for less frequent, shorter-duration, and less-severe events compared to exposed stone. In this way ivy reduces the risk of physical deterioration by sub-critical thermal fatigue and freeze-thaw weathering. Using laboratory simulations we have shown this to be the case; the thermal buffering influences of ivy translate to reductions in actual rates of deterioration measured as mass loss, surface softening and surface roughening. This effect is also likely for other types of vertical greenery that retain their foliage during the coldest winter months.

One important question still to be addressed is the extent to which thermal buffering by wall vegetation is scalable under different freezing (and heating) scenarios. There is some evidence to suggest that the greatest protective benefit will occur during the most extreme events, even if freezing is not completely prevented. For example, the likelihood of ice-related damage to stone is reduced if the severity and duration of freezing are reduced. Monitoring during a greater range of weather conditions (such as severe cold snaps) is needed to address this, and would help identify where (geographically) frost protection using vegetation could have the greatest benefit for built heritage conservation.

Under future climate scenarios the frequency of frosts will decrease in parts of Europe, but when they do occur they may be more severe and prolonged^36^. Wetter winters are also expected to be a feature of future climate in the UK, meaning that the probability of freezing events occurring when masonry is wet is also increased^62^. When coupled with its capacity to shield walls from rain^23^, the buffering potential of ivy against frost damage may shift in the future in response to changing environmental conditions^42,63^.

Our findings add to other examples indicating a ‘bioprotective’ role of vegetation for vulnerable heritage structures via microclimate regulation^61,64,65^. This presents promising opportunities for more environmentally sustainable approaches to the conservation of historic buildings and ruins. At the same time, ivy and other woody vegetation has high potential to damage vulnerable heritage structures. A nexus of deteriorative and protective effects therefore exists requiring careful evaluation, on a site-by-site basis, before management decisions and interventions are made^8^. Whilst we do not advocate growing ivy on all walls, in instances where growth is already well-established, where resources for its removal are limited, and where a risk of damage exists from specific environmental threats like freeze-thaw weathering, our observations indicate that ivy offers one option to aid conservation. As well as further work on common plants like ivy, research could usefully explore the protective potential of other vertical greenery including green facades, green screens, green walls and other self-clinging climbers as they become more widely implemented in urban areas.

## Materials and Methods

### Test walls

Purpose-built test walls were constructed in 2007 at Wytham Woods near Oxford as part of a larger study commissioned by Historic England^8^. The long-term (last 200 years) mean annual air temperature for Oxford is 9.7 °C with a mean annual rainfall of 645.6 mm. Over the last 130 years, the number of air and ground frosts has averaged around 45 and 101 per year, respectively (Radcliffe Meteorological Station, Oxford).

**Figure 5:**
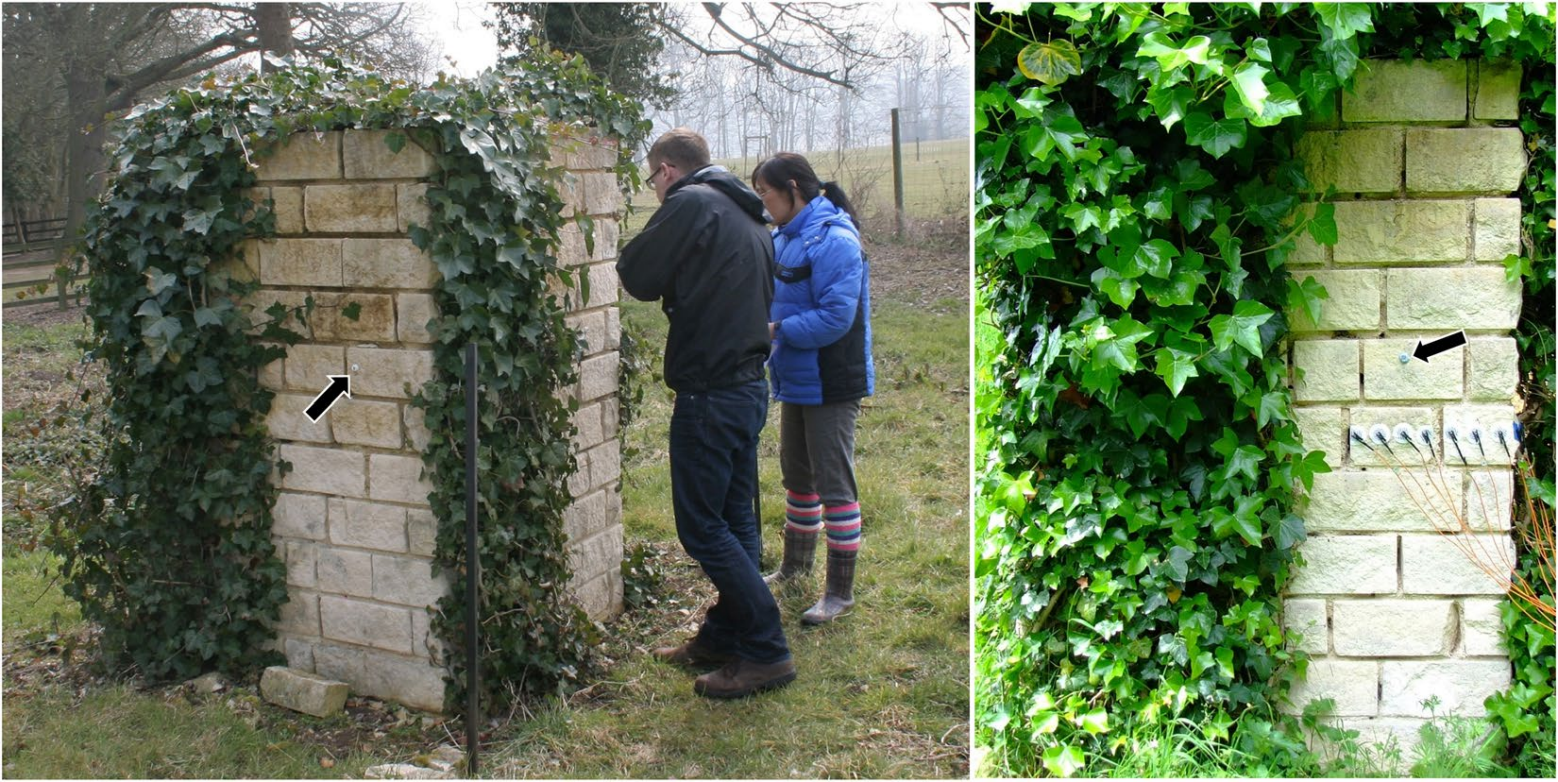
Test walls constructed of Elm Park Limestone and lime mortar, Wytham Woods, Oxfordshire. The structure had four faces (north, east, south and west facing) 1.2 m wide and 2 m high. Ivy was allowed to establish on one half of each face, allowing controlled comparisons between exposed and ivy covered stone. Arrows indicate the position of temperature loggers.

Four connecting walls were built from blocks of Elm Park Limestone (23–24 cm thick) and lime mortar around a central hollow core. The four faces were orientated north, east, south and west, respectively. Each wall face was 1.2 m wide and 2 m high (Fig. 5) and *H. helix* was planted at their bases. The walls are unobstructed on all sides to a distance of at least 10 m and prevailing winds are from the south west, although the site is relatively sheltered from the strongest winds by mature mixed woodland. In 2009 plants had become established and began to climb the wall faces. For the duration of the study, stems and foliage were regularly trimmed from one-half of each wall face. This meant that, once the ivy had reached the tops of the walls (by 2012), each test face had one half completely covered by ivy and one half completely free of growth (Fig. 5). This provided highly-controlled conditions of adjacent ‘ivy covered’ and ‘exposed’ sections on walls.

Following similar procedures to Sternberg *et al*.^26^, iButton^TM^ data loggers (DS1923, Maxim Integrated Products, ±0.5 °C) were used to monitor microclimate on the test walls. Loggers were attached to the middle of each wall (1 meter from the ground) using putty, in ivy covered and exposed pairs. Loggers were in place by winter 2012, and recorded absolute temperature at the wall surface continuously, at hourly intervals. Data collected during the winters of 2012/13 and 2013/14 were the focus of this study.

Temperature records were interrogated to determine the (1) frequency, (2) duration and (3) severity of all individual freezing events on each wall aspect, for both ivy covered and exposed areas. Frost ‘frequency’ was determined as the number of recorded fluctuations below zero degrees. Given the hourly-resolution data, these calculations represent a probable underestimate of the number of freezing events. Frost ‘duration’ was quantified as the cumulative total number of hours below zero degrees for each freezing event. Individual events were subsequently grouped into four categories (those lasting 1–5 h, 6–10 h, 11–15 h and >15 h) for analysis. The ‘severity’ of all individual frost events was calculated as the minimum temperature attained. Severity data were categorised into three groups (0 to −1 °C, −1 to −2 °C and <−2 °C). Categorical data were analysed using Chi-square tests to compare the nature of frost events between (1) exposed and ivy covered sections of wall, and (2) wall aspect. Event data were compared using Student’s t-tests for exposed/ivy covered pairs.

### Weathering simulations

An environmental chamber (Sanyo-FE 300 H/MP/R20) was used to test whether the magnitude of frost buffering by ivy recorded at the test walls had the capacity to reduce stone deterioration rates. Using laboratory weathering simulations has several advantages: experimental regimes can be designed to reflect field conditions^66^; particular variables of interest can be isolated and precisely controlled (temperature, moisture application, lithology etc.); and weathering simulations can be run in accelerated time allowing study of ‘fatigue’ modes of breakdown^67^ that may be difficult to study directly in the field.

The same stone type used to construct the test walls (Elm Park) was used for the weathering simulations. Elm Park stone is a Middle Jurassic oolitic limestone from the Cotswold Hills, with an indicative bulk specific gravity of 2245 kg m^−3^, compressive strength of 28.3 MPa (BS EN 1342) and open porosity of 17.3% (EN 1341/BRE 141). Ten 5 cm cubes were cut from the same source block to minimise structural/mineralogical variability between replicates, and the orientation of bedding-planes was standardised throughout the experiments. Prior to the start of the simulations, samples were washed to remove loose debris, oven-dried at 60 °C to constant weight, cooled in a desiccator and weighed (±0.01 g).

**Figure 6:**
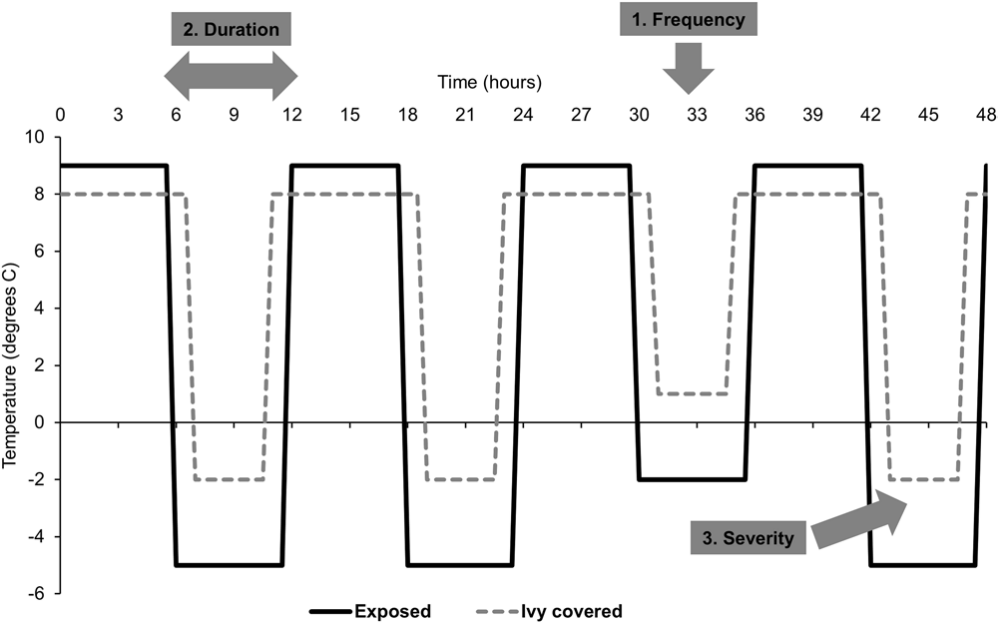
Experimental regimes used to simulate frost weathering in the laboratory. Two regimes were used representing ‘exposed’ and ‘ivy covered’ stone, accounting for ivy’s thermal blanketing as measured in the field. Adjustments were made for the frequency, duration and severity of freezing events, as indicated (also see Table 3).

Two separate frost weathering simulations were performed: the first simulated winter freezing without ivy (*exposed stone*); and the second simulated winter freezing under a canopy of ivy (*ivy covered*). A harsh but realistic regime was used for the ‘exposed stone’ experiment (cycling from −5 °C and +9 °C, Fig. 6) symptomatic of the most extreme 25% of 100 freezing events recorded in central Oxford in winter 2012/13 (Radcliffe Meteorological Station). For the ‘ivy covered’ experiment, the regime was adjusted to account for the thermal blanketing effects measured at the test walls; adjustments were made for the frequency, duration and severity of freezing, shown schematically in Fig. 6 and summarised in Table 3. The laboratory experiment was run in double-time, meaning that two ‘diurnal’ freeze-thaw cycles were simulated per 24 hours (Fig. 6). Half of the samples (*n* = 5) were subjected to the exposed stone regime and the remaining half (*n* = 5) to the ivy covered regime. Additional control samples kept under laboratory conditions were also measured for comparison.

#### Indicators of breakdown

Sample mass was re-measured after the simulations following the same procedure described above. Surface hardness was measured before and after using an Equotip3 device (with D probe) to detect changes in weathering state^68^. For this, fifty measurements were taken in a grid across the upper face of each replicate cube, taking care to avoid edge effects^69^. Qualitative observations of entire surfaces of the cubes were made using light microscopy (Leica MZ10F). As a further assessment of material change, three-dimensional topographical models were produced using an optical profilometer (TRACEiT®) for visual comparison, and from which surface roughness (Ra) was determined for five 5 mm traces on each cube (see Fig. 3).

#### Moisture application

Use of saturated samples in frost weathering experiments can lead to exaggerated results^70^. Moisture (distilled water) was therefore applied to the (initially dry) stone samples using a spray bottle. Water was sprayed directly onto the upper face of each cube at the same time of day, during the ‘thaw’ phase of the cycle. Two types of rainfall event were simulated based on historical wintertime data for Oxfordshire (Met Office MIDAS database, Brize Norton, Oxfordshire): an ‘extreme’ event reflecting the 85^th^ percentile was simulated once a week (roughly 1 L m^−2^ h^−1^) and a less extreme event based on the 60^th^ percentile was simulated four days a week (roughly 0.4 1 L m^−2^ h^−1^). No moisture was applied on the remaining two days of the week. Moisture application was therefore representative of damp winter-time conditions within the bounds of meteorological extremes recorded in Oxfordshire. Importantly, this ensured that the timing and volume of moisture application was exactly the same for the exposed stone and ivy covered stone simulations. In this way the experiment intentionally replicated the influence of ivy on thermal regimes alone, in isolation of any influences the plant has on moisture delivery – which was not the focus of this study. Both simulations were run, independently, for 6 weeks equating to roughly 85 freeze-thaw cycles for the ‘exposed stone’ regime and 63 cycles for the ‘ivy covered stone’ regime. These values fall within the range of ground frost days recorded each year in southern England (UK Met Office, www.metoffice.gov.uk/ accessed October 2017).

Differences in the weight and surface hardness of stone before and after the simulations were compared for both sample groups (exposed stone and ivy covered) using paired t-tests. Differences were further compared between the two simulations using two-sample t-tests after testing for equal variance using the F-test. Surface roughness (Ra) of samples and control cubes was also compared using ANOVA.

### Data Availability

The datasets generated and analysed during this study are available from the corresponding author on reasonable request.
